# Speechlessness: a Conceptual Framework

**DOI:** 10.1007/s12124-023-09789-6

**Published:** 2023-07-04

**Authors:** Thilo Dietz, Vera Schiewer, Ute Karbach, Michael Kusch

**Affiliations:** 1 Department of Internal Medicine I, Faculty of Medicine, Cologne University Hospital, University of Cologne, Kerpener Straße 62, 50937 Köln, Germany; 2 Faculty of Medicine and University Hospital Cologne, Institute of Medical Sociology, Health Services Research, and Rehabilitation Science, University of Cologne, Eupener Straße 129, 50933 Köln, Germany

**Keywords:** Silence, Emotions, Process model, Literature review, Non-pathological, Humans

## Abstract

The phenomenon of speechlessness has hardly been considered in the literature from a psychological point of view. Previous research on speechlessness is limited to the fields of neurology, medicine or psychopathology. The present review aims to consider speechlessness from a psychological perspective distinct from pathology, and to highlight its observability and possible connections to existing research in the context of emotional cognition and processing. Search terms were developed and a comprehensive, systematic literature search was conducted in various databases based on previous scientific work on the understanding of non-speech, silence and speechlessness. Only results that examined the phenomenon of speechlessness from a non-pathological or non-neurological perspective were included. A total of *N* = 7 publications matching the inclusion criteria were identified. The results were used to develop a procedual model for the phenomenological definition of speechlessness. The developed model differentiates the observable phenomenon of speechlessness into a non-intentional, unconscious form and a intentional, conscious form. The present work suggests that meaningful emotions and their perception and processing is a core element in the emergence of speechlessness and provides a first, psychological, non-pathological explanation of speechlessness.

## Introduction

“*Ein jeder, weil er spricht, glaubt, auch über die Sprache sprechen zu können*.” (Engl. *Anyone who speaks believes that he can speak about the subject of language as well*.) – Johann Wolfgang von Goethe, German poet 1749–1832

Speech is the primary element of human expression. Written or oral language, which is shaped by cognitive, emotional and intentional processes (Diessel, 2009; Gala et al., 2015; Levelt, 1989, 1993, 1999, 2001), conveys our feelings, narratives, descriptions, stories or information (Lingsom, 2008). The process of speech production has been described by a variety of scientific disciplines, including linguistics, psycholinguistics, neuropsychology and neuroscience (Hickok, 2014). Each of these fields offers its own claim about the process of speech production. However, comparatively few theories and concepts explore the absence of speech, also known as speechlessness.

The act of speaking involves cognitive and motor processes that encode an individual’s intentions, thoughts and emotions into acoustic signals that are decoded by a recipient. The process of speech production starts with the *conceptualisation* (meaning and purpose of what is to be said) (Levelt, 1989, 1993, 1999, 2001). In a second step, the preverbal message activates syntactic processes that create a surface structure, which in a third and final step, develops into a pronounceable phonetic plan (Levelt, 1989, 1993, 1999, 2001; Postma, 2000). In addition, the formulation process can be divided into (a) *a syntactic planning level* and (b) *a phonological planning level*.

Neurologically, the Wernicke-Geschwind model (Geschwind, 1965a, b, 1970) provides an explanation of the speech process. The model is based on the 161-year-old observations of the French surgeon Paul Broca, who identified the influence of injured brain regions on the ability to speak (Tremblay & Dick, 2016). In the Wernicke-Geschwind model, perceived speech (i.e. the auditory signal) is recorded and transmitted to Wernicke’s area^1^, which decodes the phonetic message. If the individual responds to what has been perceived, an articulatory programme is activated in Broca’s area^2^, which activates the speech muscles of the larynx (Blank et al., 2002; Price et al., 2011).

The Wernicke-Geschwind model has linguistic and neurological limitations (Hickok, 2014; Poeppel & Hickok, 2004; Tremblay & Dick, 2016). A novel approach that addresses these limitations and reflects the current state of research is the dual-stream model of Hickok and Poeppel (Hickok & Poeppel, 2000, 2004, 2007). The model describes two streams: a *ventral stream* for mapping sound to meaning and a *dorsal stream* for mapping sound to motor processes and articulation (Tippett et al., 2014). Key elements of the model are cognitive links between the two streams (*ventral* and *dorsal* stream), involving different brain regions in the speaking process. The *ventral stream* involves the spectrotemporal analysis of speech and the mapping of phonological and conceptual representations. The brain areas involved in the *ventral stream* are the primary auditory cortex, the mid-to-posterior superior temporal gyrus, the superior temporal sulcus and the mid-to-posterior middle temporal gyrus (Hickok, 2022). The *dorsal stream* supports speech production by mapping phonological representations from the left posterior superior temporal gyrus and motor speech codes in the frontal lobe (Hickok, 2022). In contrast to the classical model, the mapping of the two functions is not a direct, single-stream process, but occurs through a transformation within the posterior Sylvian region. Because of its structure, the dual-stream model explains why people with *aphasia* (inability to understand or formulate speech or language) may show only very mild symptoms of language dysfunction (Baker et al., 1981; Hickok, 2022).

Presented theories and models of language production suggest the complexity of the (verbal) language process and illustrate the complexity of this process and the ability or inability to speak. The vulnerability of this neurological process is highlighted by studies (Duffau et al., 2002, 2003) of brain plasticity. Acute injury to (specific) brain regions (e.g. a severe stroke) can lead to a loss of language comprehension and is referred by the term of *aphasia* (Damasio, 1992). Significant limitations at all levels of the language process (i.e. comprehension, reproduction, reading and writing) are summarized under the term *global aphasia* (Dronkers & Baldo, 2009). Aphasia can also occur in less severe forms such as *Broca’s aphasia* and *Wernicke’s aphasia* (Dronkers & Baldo, 2009). *Broca’s aphasia* has limitations at the level of phonological planning and is associated with anterior lesions in Broca’s area (Weismer, 2006). Patients with this form of *aphasia* can understand the content/meaning of perceived speech to a large extent (Dronkers & Baldo, 2009). Individuals with *Wernicke’s aphasia* usually have posterior lesions in Wernicke’s area that do not affect the motor speech process but the phonological representation of the person (Weismer, 2006). Despite fluent pronunciation, these individuals are often unintelligible because the words and sentences they utter cannot be understood due to incorrect intonation or lexically non-existent words (Dronkers & Baldo, 2009). There are also isolated correlations in the literature (Laganaro, 2005; Wilshire & Nespoulous, 2003) between *aphasia* and the levels of the speech production process (see Levelt, 1989, 1993, 1999, 2001). An influence of *aphasia* is found especially at the level of *phonological encoding* (subprocess of the phonological planning level of the speech process; cf. Levelt, 1989, 1993, 1999, 2001), where an increased frequency of syllable frequency errors is observed (Laganaro, 2005).

Besides neurological speech impairments, psychopathological phenomena can also lead to reduced or absent speaking behaviour. *Selective mutism* is a phenomenon of speechlessness, primarily observed in children, prototypically manifested by a complete loss of verbal communication in certain situations (e.g., school), while in other situations the child’s normal language ability is unrestricted (Muris et al., 2021). The prevalence of *selective mutism* is relatively low, ranging from 0.06 to 1.9% depending on the study and study design (Brown & Lloyd, 1975; Fundudis, 1979; Kopp & Gillberg, 1997; Kumpulainen et al., 1998).

*Alexithymia* is another pathological concept of being at a loss for words and belongs to psychosomatics. *Alexithymia* (prefix *á*- [‘*not*’], word stems *léxis* [‘*words*’] and *thȳmós* [*‘feeling*’]) means “*the inability to recognize one’s own feelings and to express them, especially in word*” (Oxford English Dictionary, 2021). The term was coined by the authors Nemiah and Sifneos (1970) and has its origins in psychosomatic medicine. Individuals have difficulty perceiving, identifying and describing emotions (Bagby & Taylor, 2020; Preece et al., 2017). Individuals also struggle to distinguish physical sensations from emotional responses (Taylor, 2000) and are more likely to experience major depression (Honkalampi et al., 2001), anxiety (Zeitlin & McNally, 1993) and personality disorders (Berenbaum, 1996). Furthermore, there is evidence that alexithymia has a negative impact on a person’s quality of life (Leenen et al., 2013). The proportion of lack of communication in therapy sessions with alexithymic patients has been studied by Overbeck (1977), whose findings indicate that, on average, up to 40–50% of the time in therapy sessions is spent in silence.

The phenomenon of inability to pronounce the first name of an interaction/interlocutor, described with the term *alexinomia* (prefix *á*- [‘*not*’], word stems *léxis* [‘*words*’] and *ónoma* [‘name’]), serves in identical form the concept of alexithymia in loanwords of the Greek language (Ditye et al., 2023). First references to this phenomenon in the scientific literature can be found in Welleschick (2019), however, the researchers centred around Ditye et al. (2023) are the first to address the phenomenon from a psychological perspective. The phenomenon often begins in childhood. Potential factors predisposing individuals to alexinomia include social anxiety disorder, childhood trauma, negligence, and other personality disorders in the individual’s family (Ditye et al., 2023). The authors note, that affected individuals “*have a desire to use personal names in everyday social interactions*, (…)”. (Ditye et al., 2023, p. 13), but are impaired in their execution. The onset of the phenomenon is also accompanied by feelings of anxiety, panic, and psychological and physiological discomfort (Ditye et al., 2023). The question of whether alexinomia is a psychosomatic or psychopathological phenomenon has not been discussed in the literature.

In addition, a loss of the ability to speak may be triggered by a traumatic experience. A trauma is characterized by an extraordinary threat or catastrophic event (Dilling et al., 1991), which may involve a confrontation with one’s own death (American Psychiatric Association, 2013). Depending on the intensity of the trauma experienced, this can be a lasting (chronic) burden for the individual (cf. *post-traumatic stress disorder*). Laub (2005) describes the attempt of Holocaust survivors to describe their own experiences of horror, which resulted only in “*moaning*” or “*barely audible*” screams (Laub, 2005, p. 259). Parallels can be found with chronically ill individuals, who also describe their condition and its treatment as a traumatic experience and therefore shroud themselves in silence (Penn, 2001). Van der Kolk (1987) elaborates on this approach and defines an experience beyond human imagination as an experience of “*speechless terror*”. The experience is so overwhelming that an expression in linguistic form fails due to a missing link between memory and words (Harris, 2009; van der Kolk, 1987; van der Kolk et al., 1996; van der Kolk & van der Hart, 1991).

Psychopathology, in particular, significantly shapes society’s perception of an affected person (Ahmedani, 2011). Individuals with mental illness are perceived by the public as being of unsound mind, rejected (Sendera & Sendera, 2012), and discriminated against in employment and housing (Corrigan et al., 2004; Hinshaw & Cicchetti, 2000). There is also a lack of care and health insurance for stigmatized individuals (Holmes et al., 1999; Torrey, 1997). Affected individuals are stigmatized (World Health Organization, 2001), sometimes even considered abnormal (Raskin, 2019) if they are physically or mentally ill, behave differently, relate disharmoniously to society, or lack the ability to communicate with others (Arboleda-Flórez & Stuart, 2012). In this context, the distinction of the mental state into healthy or ill is subject to the scientific and social “*Zeitgeist*” (Sendera & Sendera, 2012, p. 1). Mental disorders can be perceived as “*punishment for sin*” (Adshead, 2008, p. 423), as illness or psychopathology (Raskin, 2019), or as a response to stress and adversity that can be meaningful (Clifford & Lemery-Chalfant, 2015; Davydov et al., 2010; Read & Harper, 2022). It should therefore be emphasized that, in the context of this work, speechlessness is not seen as another mental illness or disorder, but as an unusual but meaningful response to an outstanding but personally relevant adversity.

A lexical (non-pathological) definition of speechlessness describes it as a person’s reaction caused by a state of shock or extreme emotion (Guralnik, 1980). Within this definition, emotional perception and processing is a core element of speechlessness, distinguishing it in this respect from the neurological and psychopathological forms of speechlessness mentioned above. As noted in the introduction, the ability of verbal language has been extensively studied by various scientific disciplines. However, the absence of verbal language has been studied exclusively from a pathological point of view. Nevertheless, we encounter the phenomenon of speechlessness almost every day. This review aims to present a description and concept of the phenomenon of speechlessness from a psychological perspective, separated from neurological, psychosomatic and psychopathological definitions. Based on this approach, speechlessness is considered an observable phenomenon, caused by intrinsic motivations or external factors. Moreover, the approach is to illuminate speechlessness as a complementary field of research and to identify potential interconnections with existing research. Based on preliminary work and a subsequent literature review, speechlessness is examined in the context of emotional perception and processing.

## First Investigations on the Phenomenon of Speechlessness

The scientific literature has hardly dealt with a psychological construct or phenomenon of speechlessness apart from the neurobiological, psychosomatic and psychopathological types of speechlessness discussed earlier. Berger (2004, 2015) is one of the few authors who has empirically explored speechlessness as a psychological and linguistic concept. He defines speechlessness as “*an involuntary state*” (Berger, 2004, p. 173) that exceeds the normal duration of a conversational pause and impedes communication between interlocutors^3^. He summarized the five most common causes of speechlessness based on empirical data: (1) *unexpected information / deviant behavior*, (2) *stress, extreme emotions or nervousness*, (3) *lack of information / knowledge about topic*, (4) *uncertainty about own/ other’s feeling*, (5) *difficulty answering unanticipated questions* (Berger, 2004, p. 154). His findings also suggest that silence or speechlessness is caused by another person’s actions and words, intense emotional states, lack of knowledge, and fear of feelings such as guilt or shame. In terms of the variety of events that can trigger speech loss, fear was the most important emotion experienced by individuals. Berger (2004) further concludes that in some of the situations recorded, it was possible for those affected to say something, but they chose not to. These situations are therefore classified as *strategic speechlessness* a “[…] *strategy for dealing with problematic social situations.*, […].” (Berger, 2004, p. 171). On the other hand, *non-strategic speechlessness* is primarily framed in terms of violated expectations and extreme emotions (Berger, 2004). Several other authors adopted this two-dimensional classification of speechlessness into a *strategic* (synonym: *volitional* or *intentional*) and a *non-strategic* (synonym: *non-volitional* or *non-intentional*) component (Ephratt, 2008; Kurzon, 2007, 2020; Stringer et al., 2010). In his work on the functions and forms of silence in social interactions, Kurzon (2007) refers to Berger’s classification of strategic and non-strategic speechlessness. However, he applies silence as a synonym for speechlessness (“[…] *students* […], *who were asked to recall the last instance of silence they remembered*”; Kurzon, 2007, p. 1675) while Berger (2004) writes in the original “[…] *participants were asked to recall the most recent social episode during which they experienced speechlessness or were at a loss for words.*“ (Berger, 2004, p. 150).

Silence and speechlessness can be used synonymously, as they are indistinguishable from each other (Kurzon, 1998, 2007, 2020). Bruneau (1973) defines three forms of silence: (1) *psychological*, (2) *interactive*, and (3) *sociocultural silence*. While *psychological silence* can be attributed to an unconscious, physiological process (e.g. stuttering or hesitating to answer), *sociocultural silence* forms a latent factor that “*manipulates*” *psychological* and *interactive silence* (Bruneau, 1973, p. 36). Bruneau (1973) regards *interactive silence* to be in the context of interpersonal relationships, which can be seen as a conscious process in conversations. Kurzon (2007) takes these assumptions a step further and proposes four types of silence: (1) *conversational*, (2) *thematic*, (3) *textual* and (4) *situational silence*. Both, *conversational* and *thematic silence* are types of silence in the context of a social conversational situation, whereas *textual silence* refers to situations in which an individual chooses to read (e.g. library) or pray (e.g. church) in silence. In contrast, *situational silence* refers to silence in larger groups of individuals that take place in the context of a social situation (e.g. participation in a public memorial with a moment of silence). The understanding of silence as speechlessness is based on definitions found in the literature. Kurzon (2020) defines non-intentional silence as a psychological origin that arises when an individual is “*shy*” or “*embarrassed*” (Kurzon, 2020, p. 50). This may occur when they do not know the answer to a question and prefer to remain silent rather than admit their inability to answer (Kurzon, 1998, 2020). Intentional silence, on the other hand, can be expressed with a goal of an individual to not answer a question regardless of internal or external factors (Kurzon, 2020). Kurzon (2020) cites the Sicilian Mafia’s code of silence (omertà^4^) as an example of the distinction between intentional and unintentional silence. Internal causes for intentional silence are “[…] *personal reasons*, […]” (Kurzon, 2020, p. 50). Examples for an external cause of intentional speechlessness are situations, where another person is “*threatening*” the silenced individual (Kurzon, 2020, p. 50). Silence, however, is hardly distinguishable from non-strategic speechlessness for the interlocutor. The silence of the interlocutor forms a temporary state following the sequential sequence of utterances of the common conversational situation (Kurzon, 1995, 1998). Moreover, it can be used in the context of a conversational situation as a conscious process of the speaker’s embodiment of his or her own emotion (cf. *emotive functions*, Ephratt, 2008).

Table 1 provides a comparison of the different forms of speechlessness depending on the discipline under consideration.

**Table 1:** Definitions of speechlessness depending on scientific discipline

Scientific discipline	Name	Definition	References
Neuro-biological	Aphasia	Disorder of language comprehension and development caused by cognitive dysfunction or trauma	(Damasio, 1992)
Psychopathological	(Selective) Mutism	Partial or complete absence of speech in specific situations (e.g., school), although a normal speech ability is presented/unrestricted	(Muris et al., 2021)
	Speechless Terror	Traumatic memories that occur throughout life and, due to cognitive processes during the traumatic experience, cannot be completely realized and accurately described later in life.	(Harris, 2009; van der Kolk, 1987; van der Kolk et al., 1996; van der Kolk & van der Hart, 1991)
Psychosomatic	Alexithymia	Dysfunction in the perception, identification and (verbal) expression of emotions.	(Bagby & Taylor, 2020; Preece et al., 2017; Taylor, 2000)
Psychological	Speechlessness	Non-strategic or strategic non-speaking that lasts beyond the duration of a normal pause in speech and impedes communication between interlocutors	(Berger, 2004)
	Silence	Different forms of silence depending on contextual factors; primarly types of speechlessness to be adressed: (1) psychological silence can be attributed to an unconscious and physiological processes; (2) sociocultural silence forms a latent social factor that leads to silence (3) interactive silence is an interactive, conscious process during conversation	(Bruneau, 1973)
		(1) conversational silence often occurs in dyadic interaction and can be considered as equivalent to the act of speech (2) thematic silence ccours in a conversational context where the individual does not say something regarding a specific subject (3) textual silence refers to a specific context, where an individual reads in silence (e.g. library or at home) (4) situational silence occurs in situations, where larger groups of people do not speak relating to situation or context (e.g. church or memorial)	(Kurzon, 2007)
	alexinomia	Inability to pronounce a person’s personal name in social interactions and/or interpersonal relationships between multiple people.	(Ditye et al., 2023)

## Systematic Literature Search

Referring to Berger’s definition of speechlessness, it becomes clear that it is based on a non-strategic character, primarily driven by negative-valued emotions (e.g. shame or embarrassment). From an observational perspective, however, it is difficult to distinguish between non-strategic speechlessness (cf. Berger, 2004) and intentional silence. Moreover, parallels can be found between the two forms of non-speech. These parallels can be discussed primarily in terms of emotional perception and/or processing (cf. Ephratt, 2008). According to these parallels, a combined review of the literature focusing on studies of the phenomenon of speechlessness from the perspective of non-strategic and strategic non-speaking is necessary.

In order to describe the phenomenon of speechlessness from a psychological, non-pathological point of view and to combine the above parallels, an initial framework of speechlessness was developed. This model was derived from the literature on speechlessness or lack of speech that was identified in the *PubMed* and *APA PsycINFO* databases, using a total of five search terms (Table 2). Peer-reviewed research papers and studies on speechlessness and silence with empirical or exploratory characteristics in adults, published from the earliest available record to November 2022, were included. Studies investigating speechlessness and silence based on neurobiological (e.g. aphasia), psychosomatic (e.g. alexithymia) and psychopathological (e.g. [selective] mutism) causes were excluded from the analysis. Dissertations, scientific posters and congress contributions as well as studies in languages other than German or English were also excluded. The screening and selection process were performed independently by the first and second author (TD and VS). The inclusion of relevant literature was done step by step. Studies with titles that did not appear to relate to speechlessness/silence were excluded. In a second step, abstracts of studies with titles clearly related to speechlessness/silence or studies with unclear titles were screened. Abstracts and titles were read independently by both authors in order to determine whether the articles would be suitable for inclusion in the review. If it were not possible to include or exclude a study based on the abstract, the full text was assessed before the final decision was made.

**Table 2:** Search terms

Used database	Search term	Number of literature identified using the search term	Number of identified literature included in the full-text analysis	Number of previously identified literature which, after detailed analysis, uses a non-pathological term for speechlessness
APA PsycINFO	( (“psychological silence” OR “interactive silence”) AND (“speechless” OR “speechlessness”) ) NOT mutism NOT selective mutism NOT aphasia	19 (18.4%)	1 (8.3%)	1 (14.3%)
	( (“conversational silence” OR “thematic silence” OR “situational silence”) AND (“speechless” OR “speechlessness”) ) NOT mutism NOT selective mutism NOT aphasia	14 (13.6%)	2 (16.7%)	0 (0%)
	“volitional speechlessness” OR “strategic speechlessness” OR “non-volitional speechlessness” OR “non-strategic speechlessness”	1 (1%)	1 (8.3%)	1 (14.3%)
PubMed	(((speechless OR speechlessness) AND (emotion OR emotion regulation*)	10 (9.7%)	1 (8.3%)	0 (0%)
	(((speechless OR speechlessness) NOT (mutism)) NOT (selective mutism)) NOT (aphasia) AND (humans)	59 (57.3%)	7 (58.3%)	5 (71.4%)

Since PubMed is primarily a medical database and APA PsycInfo is focused on psychological publications, the search terms between the two databases differ

*Note.* The percentages in parentheses refer to the total number of identified/reviewed publications per table column; N = Number of articles

### Results

**Fig. 1:**
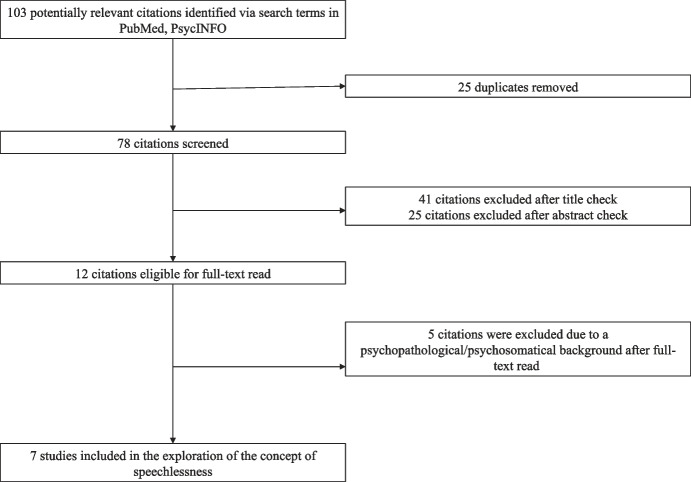
Flow chart

The literature search identified a total of *N* = 103 potential articles. After title and abstract screening, *N* = 12 full texts were read. Five (42%) of the 12 articles addressed speechlessness/silence in the context of psychopathological (e.g. trauma, borderline personality disorder, anorexia nervosa; depression and/or anxiety disorder) and/or psychosomatic (e.g. alexithymia) concepts. In addition, the work of Berger (2004), already cited in the introduction of this work, was extracted and included in the literature review. A total of seven articles (58%) included a description of speechlessness and/or silence in accordance with the aim of the systematic literature review (Fig. 1). The included articles were assessed by the first author (TD) and classified as follows: (A) *type of work*, (B) *study design and context of speechlessness/silence*, and (C) *in-text definition* (Table 3).

Five of the seven studies (71%) represent an empirical investigation of speechlessness. Only one study (Berger, 2004) collected quantitative data, the remaining studies had a qualitative focus (Fabiane & Corrêa, 2007; Koskinen et al., 2016; Kummer et al., 2008; Lillrank, 2002). Two identified literature sources (Kahn, 2019; Müller-Cyran, 2007) are case studies/commentaries.

**Table 3:** Definitions of speechlessness in the identified literature

Type of work	Study design / context of speechlessness and/or silence	In-text definition	Author / Year
Quantitative study	Study 1: A total of n = 210 students participated in a questionnaire survey and received prior information about speechlessness and its occurrence. Participants were asked to recall the last situation in which they felt speechless. Study 2: A total of n = 120 students were presented with a modified version of the questionnaire used in Study 1; however, identical information and instructions for completing the questionnaire were retained. In addition, students were asked to rate a list of 18 potential reasons for speechlessness on a three-point Likert scale.	Study 1: “Apparently, participants did not spontaneously think about the causes of their speechlessness at this level of detail. Analyses of the three most frequently mentioned loci suggest a number of plausible alternative sequences that result in speechlessness, as well as differences in the emotions associated with each sequence.” (p. 160) “Analyses of the three most frequently mentioned loci suggest a number of plausible alternative sequences that result in speechlessness, as well as differences in the emotions associated with each sequence. Although a multitude of events might prompt extreme emotional states and stress, the primary emotion driving the speechless event was fear, not surprise, and once speechlessness ensued, individuals reported being less confused than those designating other causes.” (p 0.161) Study 2: “Although recalled instances of speechlessness again favored those that were involuntary and participants attributed their speechlessness more to their inability to find words to express their intentions rather than their inability to express conceptually formulated messages in speech, individual comparisons of voluntary and involuntary speechlessness attributions with these loci of encoding difficulty failed to produce significant relationships. Because the means of the two encoding difficulty items and some of the involuntary speechlessness loci were quite high and because several of the voluntary speechlessness loci were rated quite low, the restricted ranges of the scales may have attenuated potential correlations between them. Thus, support for the first hypothesis remains circumstantial.” (p. 171) “The results not only demonstrate that different causal loci for speechlessness are associated with different affects, they also show that violated expectations and extreme emotional states in particular are likely to induce more negative affect than are the other causal loci, including those associated with strategic silence.” (p. 171) General Discussion: “The results of these initial investigations of speechlessness present a complex picture of the phenomenon.” (p. 172) “Such attributions as unexpected behavior, another’s overly intense behavior and extreme emotions suggest that expectation violations play an important, but not exclusive, role in potentiating speechlessness; thus, speechlessness may be implicated in interpersonal adaptation process (Burgoon et al., 1993; Guerrero et al., 2000). Specifically, speechlessness may at once be an indicator of violated expectations and, once precipitated, may require significant interpersonal adaptations for its management.” (p. 173) “If instances of strategic silence are excluded from the domain of speechless events, the present studies suggest that speechlessness or being at a loss for words is an involuntary state that renders interlocutors unable to produce speech for periods of time that exceed the usual durations of nonvocalized pauses in fluent speech.” (p. 173)	(Berger, 2004)
Qualitative study	The setting of the survey was an intensive care unit with ten beds for the intensive care of patients with various diseases, excluding trauma and/or infections with multi-resistant pathogens. The respondents were relatives of the hospitalized patients. Subjects of the survey formed the nuclear family (father or mother; child; relatives, married or in partnership). A total of 17 interviews were conducted and recorded. The entire interview was conducted considering one guiding question (What is it like for you to have a relative hospitalized here? Please explain).	“Many relatives, despite their initially expressed difficulty and fear, report that they cannot find a specific word to express the painful situation they are living. During the interviews, it was clear that they tried to find, by gesticulation and silence, a word that would express their feelings, but they were not able to. The feeling was stronger than what could be expressed in words, since words sometimes do not translate the experienced situations.” (p. 600)	(Fabiane & Corrêa, 2007)
Commentary / case description	First case/situation: A young, female patient in a psychiatric facility tried to commit suicide with gloves knotted around her neck. The author describes his perceptions and behavior in this situation. Second case/situation: The author describes dealing with a delusional patient who believes that a microchip is implanted in his molar. The patient insists on performing diagnostic measures (CAT-Scan / X-rays) to prove his version of reality (based on psychosis) and wants to have the molar removed. Third case/situation: The author generally describes the management of bigoted and sexually provocative patients.	First case/situation: ”A clinician in such situations has to say something — but what? Stricken with acute speechlessness, I knew that at the very least I didn’t want to swallow Lisa’s proffered bait and retaliate punitively.” (p. 507) Second case/situation: “He had made his request and waited for my answer. I had nothing to say for at least a few seconds, although it felt much longer. Then it dawned on me to just say what was inarguable: ‘Bill, here’s how I see things. I’m afraid I don’t think the chip is there but I know I can’t convince you of that, and I won’t even try. If I can be straight with you, I think your imagination is getting the better of you on this one.’” (p.508) Third case/situation: “Possibly the most common and malignant variety of clinician speechlessness is that caused by bigoted or sexually provocative patients. Here the doctor’s lack of words is complicated with shock and outrage, rather than with the anxiety of the previous examples.” (p.508)	(Kahn, 2019)
Qualitative study	Recruitment of patients through pain association leaders. Four patients from the same pain association were invited to participate in the study; these patients constitute the study population. Data collection by focus group interview. The interview was conducted and recorded in a semi-structured format. Interviews were transcribed and analyzed using qualitative content analysis.	“Protracted pain may be difficult to describe in words, and it can be hard to find words to describe all the dimensions that the pain entails. Not even swear words are enough to describe the pain. Pain can take the words out of a human being’s mouth and render him or her speechless.” (p. 777)	(Koskinen et al., 2016)
Qualitative study	Data collection in a geriatric rehabilitation clinic. Patients constituted incontinent geriatric patients (> 59 years). For data collection, guided interviews were developed on a prior observation of the patients’ care. Three groups consisting of affected patients, nursing staff, and medical staff were interviewed.	“Nevertheless, there are areas of treatment where speechlessness seems to prevail between professionals and patients, at least experts state […]: Incontinence is one such condition that seems to leave interlocutors sitting speechless across from each other.” [original citation: „Dennoch, es gibt Behandlungsbereiche, da scheint Sprachlosigkeit zwischen den Professionellen und Patienten zu herrschen, so konstatieren zumindest Experten […]: Inkontinenz ist eine solche Erkrankung, die Gesprächspartner sprachlos einander gegenüber sitzen zu lassen scheint.“ (p. 268)] “Instead of allowing speechlessness to prevail, a communication bridge - verbal as well as non-verbal - needs to be built with patients.” [original citation: „Statt Sprachlosigkeit walten zu lassen, muss eine Kommunikationsbrücke – verbal sowie non-verbal – zu Patientinnen und Patienten gebaut werden.“ (p. 272)]	(Kummer et al., 2008)
Qualitative study	Interview with parents of children with cancer (< 12 years; not hospitalized at the time of interview). Thirty-two one- or two-parent families participated in the survey. In summary, n = 49 parents were interviewed. Mother and fathers were interviewed separately. Interviews were audio-recorded and transcribed. Superordinate themes were derived based on the transcripts.	“When Maire told me her story I became overwhelmed with unarticulated feelings of exhaustive emotional pain. Her non-verbally communicated emotional pain took me by surprise because of such an overwhelming sense of anxiety, despair and helplessness […]. It unconsciously triggered my memory of a personal crisis in another social context. This enabled me to recognize the depth of her emotional pain and to contain it. Nevertheless, it overwhelmed me in such a way that I became speechless, unable or comment or ask related questions. Other possible reactions could have been to split it off immediately, or for example, deny its painfulness by reassurance […].” (p. 118)	(Lillrank, 2002)
Commentary	The author describes in detail the situation of an emergency operation with resuscitation measures, in which the affected person dies while a close person (relative) is present. The author then goes into detail about the feelings of the relative as well as the feelings of the emergency physician and operationalizes speechlessness to describe the situation.	“Another speechless experience is highly relevant to medicine because here the borderline of language coincides with a borderline of medicine: the confrontation with death.” [original citation: „Eine andere sprachlos machende Erfahrung ist hochgradig relevant für die Medizin, weil hier die Grenzlinie von Sprache mit einer Grenzlinie der Medizin in Eins fällt: die Konfrontation mit dem Tod.“ (p. 283)] “While emergency medicine is invasive and stringent, psychosocial support starts first with the bereaved person’s experience that another human being perceives and takes him seriously and shares his speechlessness, his speechless grief and helplessness - beyond words.” [original citation: „Während in der Notfallmedizin invasiv und stringent gearbeitet wird, setzt die psychosoziale Unterstützung zunächst mit der Erfahrung des Hinterbliebenen ein, dass ein anderer Mensch ihn wahr- und ernst nimmt und seine Sprachlosigkeit, seine sprachlose Trauer und Hilflosigkeit – jenseits aller Worte – teilt.“ (p. 283)] “The need for emergency medicine highlights that even the domestication of death is only partially successful. When a person dies despite the physician’s commitment, the physician is confronted with functional helplessness: The occurrence of death or the inability to stave off death cannot be blamed on him or her” [original citation: „Der Bedarf an Notfallmedizin zeigt auf, dass auch die Domestizierung des Todes nur teilweise gelingt. Wenn ein Mensch gegen das Engagement des Arztes stirbt, ist der Arzt mit funktionaler Hilflosigkeit konfrontiert: Das Eintreten des Todes oder das Unvermögen, Tod abzuwehren, ist ihm nicht anzulasten“ (p. 284)]	(Müller-Cyran, 2007)

#### Empirical Studies^5^

Fabiane and Corrêa (2007) studied the perceptions of the ICU environment and the impact of intensive care of hospitalized patients from the perspective of their relatives (nuclear family). From their data analysis, the authors concluded that many relatives had difficulties “*finding a specific word to express the painful situation they were experiencing*” (Fabiane & Corrêa, 2007, p. 600). The relatives’ speechlessness is expressed by the fact that they try to say something, but are not able to, and therefore try to find an expression for their own feelings through gestures and silence (cf. Table 3).

Koskinen et al. (2016) interviewed people with protracted physical pain. The authors describe that the affected people have difficulties to describe the dimensions of pain (“[…] *difficult to describe in words*, […]”, Koskinen et al., 2016, p. 777). The concept of speechlessness within this study, depicts a ‘not speaking’ or a ‘not being able to speak’ due to the immense pain experienced.

Kummer et al. (2008) investigated the communication over incontinence between caregivers (nursing and medical professionals) and affected patients. The authors equate the concept of speechlessness with thematic silence or topic avoidance between the two interlocutors (original citation “[…], *die Inkontinenz ist eine solche Erkrankung, die Gesprächspartner sprachlos einander gegenüber sitzen zu lassen scheint.*“ Engl. translation “[…], *incontinence is the kind of condition that seems to leave interlocutors sitting speechless across from each other.*“ Kummer et al., 2008, p. 268). Moreover, the use of the term ‘speechlessness’ can be associated with an intentional silence. The authors conclude that there is a need for communication between providers and patients, whether verbal or non-verbal (see Table 3).

Speechlessness resulting from emotional overload is described by Lillrank (2002). The author refers to a conversation with a mother of a child with cancer and describes the emotional pain that emanated from the mother. This situation evoked a memory in the author that made her feel and understand the pain. Consequently, she describes, “*it overwhelmed me in such a way that I became speechless, unable to comment or ask related questions”* (Lillrank, 2002, p. 118).

#### Non-Empirical Studies

Kahn (2019) describes three situations that left him speechless as a psychiatric care physician. In the first situation, he refers to the suicide attempt of a young, female patient and her facial expression and the author’s interpretation of it. The author describes himself as “*stricken with acute speechlessness*” in this situation (Kahn, 2019, e507). The second situation mentioned by the author, describes dealing with a delusional patient. The author portrays that the patient was expecting a response to his expressed desire for further diagnostic measures or surgical removal of his molar, but the author had nothing to say (“*I had nothing to say for at least a few seconds*, […].“ Kahn, 2019, e508). Finally, Kahn (2019) describes that probably the most commonly experienced reasons for speechlessness are from “[…] *bigoted or secondarily provocative patients.*“ (Kahn, 2019, e508).

Müller-Cyran (2007) describes speechlessness against the background of the death of a close relative after emergency medical first aid. The concept of speechlessness is operationalized in different ways. On the side of the bereaved, speechlessness is equated with “*speechless grief*” (Müller-Cyran, 2007, p. 283; cf. Table 3). Due to a dissociative experience of the situation, the bereaved person can hardly put the experience into words, similar to the emotional extremes empirically recorded by Berger’s (2004) survey. Speechlessness of the caregiver is described by Müller-Cyran (2007) more as helplessness. In these situations, the caregiver has to accept the death of the relative because their own services/life-sustaining measures have failed. This situation results in what the author calls “*the ‘speechlessness’ of the helpers*” (de.: “*Die ‘Sprachlosigkeit’ der Helfer*”, Müller-Cyran, 2007, p. 283), a concealment/silence due to one’s own incompetence.

### Interpretation

Two (Fabiane & Corrêa, 2007; Lillrank, 2002) of the seven studies name speechlessness or silence in the context of extreme emotions or extreme emotional situations. The authors’ notion of speechlessness is consistent with Berger’s (2004) reasons/situations for *non-strategic speechlessness*. In essence, the inability to speak is due to an extreme emotional situation. The speechlessness of patients with protracted pain recorded by Koskinen et al. (2016) can likewise be framed in the context of meaningful^6^ or frightening emotions. Pain and emotions are interdependent with each other (Lumley et al., 2011). Stressful situations or conflicts can lead to different pain conditions (e.g. migraine headaches, irritable bowel syndrome, fibromyalgia) (Imbierowicz & Egle, 2003; Mayer et al., 2001; Sumanen et al., 2007).

One study (Kummer et al., 2008) utilized the speechlessness term in the formal context of hospital-based care. Application of the speechlessness term is related to avoidance of a specific topic. This avoidance diverges from the defined types of silence (Bruneau, 1973; Kurzon, 2007, 2020) and the moments of speechlessness captured by Berger (2004). By avoiding the naming of a problem (specifically that of potential incontinence; Kummer et al., 2008), the acquired shape of speechlessness is to be placed in a context of *strategic silence* (cf. Berger, 2004).

Additional similarities exist in the situations of self-perceived speechlessness described by Kahn (2019). Violated expectations can be attributed to the situation and/or a surprise in the intention of his patient. The author’s silence in the second situation (cf. Table 3) can be explained by the difficulties of answering unexpected questions (cf. Berger, 2004). The third situation (cf. Table 3) described by the author can be assigned to speechlessness triggered by unexpected behavior (cf. Berger, 2004). However, the author’s silence may also be rooted in the need to formulate an appropriate response while considering various factors. This form of speechlessness parallels the concept of expressive suppression (ES), in which people suppress their emotional expression (Gross, 2014, 2015; Gross & Levenson, 1993).

## Linking Speechlessness to Psychological Theories

Based on the literature identified, similarities to Berger’s (2004) observations (a. *unexpected information / deviant behavior*, b. *difficulties answering unanticipated questions*, and c. *stress, extreme emotions, or nervousness*) were found in the literature discussed above. In addition, two other reasons for speechlessness were identified: (1) *expressive suppression* and (2) *strategic silence*. The identified causes of non-strategic and strategic speechlessness can be linked to psychological theories and concepts.

Berger’s (2004) *non-strategic speechlessness* in response to unexpected information/ behavior can be explained by several (social) psychological theories. Social norms and values (Ajzen, 1985, 1987, 1991) shape an individual’s actions and subjective expectations. Expectations form persistent patterns of behavior that individuals prefer, consider appropriate or desirable, and are shaped by the social environment (internalized norms and values; e.g. stopping at red traffic lights) (Burgoon & Walther, 1990). The influence and handling of violated expectations is addressed by the *Expectancy Violations Theory* (EVT; Burgoon, 2015). The EVT provides a contextual framework of interpersonal communication, describing the outcome expectation as well as the expectation violation of the individuals involved in the situation (Burgoon, 2015; Burgoon & Walther, 1990). The assumption of EVT is that every interaction is preceded by an evaluation of the counterpart (social status, intellect, appearance [attractiveness], etc.) and, based on this evaluation, if the interaction appears to be worthwhile. A contradictory or unfulfilled expectation is defined as an expectation violation (Burgoon, 2015), which occurs whenever there is a clear discrepancy between the behavior that is anticipated and the behavior that is expected (Floyd et al., 1999). Depending on the type of violation, an invisible threshold of coercion for the recipient (expecting individual) can be exceeded, triggering discomfort or a sense of threat in the individual. The greater the violation of expected behavior, the greater the effect (Burgoon, 2015). Whether a violation of expectations is evaluated positively or negatively depends on the reward valence of the recipient (expecting individual) and whether the invisible threat threshold is crossed (Burgoon, 2015). Regardless of the evaluation of a violation of expectations, the latter is associated with an emotional experience in the ‘violated person’ (Bennett et al., 2020). Depending on the intensity of the experienced emotion, it may cause speechlessness within the individual. This interpretation can be reproduced using the situations described above (see Kahn, 2019; Table 3).

The speechlessness associated with difficulties in responding to unexpected questions (and situations) can be explained by a lack of self-efficacy and perceived control. Berger (2004) suggests that “[…] *individuals who tend to view their task environment as uncontrollable are likely to suffer loss of self-efficacy with respect to the task* […]. *Thus, individuals whose speechlessness is involuntary should feel less control over their emotions and actions and thus feel more intense levels of negative affect.*“ (Berger, 2004, p. 174). Self-efficacy describes a person’s assessment of their own ability to perform desired actions in an organized and successful manner (Bandura, 1977, 1986, 1997). Subjective beliefs control the individual’s cognitive, motivational, emotional and actionable processes. This occurs primarily through outcome expectancies and perceived self-efficacy. The extent to which the person is able to learn these competencies is described by self-efficacy. However, if the person does not see themself as capable of acquiring these competencies, this is considered to be weak self-efficacy (Schwarzer & Jerusalem, 2002). The higher a person’s self-efficacy, the more likely that person is to demonstrate the ability to meet the demands placed on them by means of innovation and perseverance (Schwarzer & Jerusalem, 2002; Voica et al., 2020).

A complementary approach is the concept of psychological flexibility (PF), which describes an individual’s ability to maintain or change their behavior by consciously engaging with their own feelings and thoughts, making an assessment of the situation and also acting in the context of their own values and goals (Hayes et al., 2004, 2006). The concept of PF is based on research in the field of emotion regulation and consists of the assumption that individuals are motivated to experience fewer negative emotions and to experience more positive emotions (Doorley et al., 2020; Tice et al., 2004). In particular, the counterparts to the PF processes of *acceptance* (*experiential avoidance*) and *cognitive defusion* (*cognitive fusion*) are proposed as relevant factors in the emergence of speechlessness. Cognitive fusion supports the development of experiential avoidance. Experiential avoidance is associated with a change in an individual’s behavior to reduce the frequency and content of unwanted emotions and perceptions (Hayes et al., 1999), even if this behavior has negative consequences for the individual (Hayes et al., 1996, 2006). (Khalid et al., 2020) conclude that the use of experiential avoidance increases defensive silencing.

The avoidance of perceiving certain content and emotions, and the resulting silence, has parallels with the intentional speechlessness as a strategy for coping with problematic social situations (cf. Berger, 2004). Avoiding specific content is also part of topic avoidance, a goal-oriented communication behavior in which individuals strategically avoid certain topics of conversation (Dailey & Palomares, 2004). This form of speechlessness is also found in the work of Kummer et al. (2008) mentioned above. The speechlessness described by the authors is less an emotional speechlessness or a speechlessness caused by violated expectations, but rather a conscious ignoring of the demand for possible diseases/comorbidities by the healthcare professionals. There is also evidence in the literature that strategic silence stems from a conscious decision of the individual (Dimitrov, 2017). In this case, silence/speechlessness is interpreted as a communicative, dynamic process (Dimitrov, 2017). It should be added that Berger (2004) already suggests that people who were strategically silent knew what they wanted to say in certain situations but chose not to speak. He also argues that strategic silence should be distinguished from involuntary (non-volitional) speechlessness. This assessment can also be derived from the cited source (cf. Kummer et al., 2008).

Lastly, it is worth mentioning the theory of emotion regulation (Gross, 2014) as a complementary concept in psychology. This theory provides a model of the process by which emotions are perceived and evaluated. Emotions are an important mediator in the development of (non-) intentional speechlessness. The intensity, duration, and timing of emotional experience is influenced by the process of emotion regulation (Gross, 2014). Comparable to experiential avoidance (cf. Doorley et al., 2020; Hayes et al., 2006, 1996; Tice et al., 2004), emotion regulation usually occurs for socially motivated reasons, such as not wanting to hurt or harm another person (Fischer et al., 2004). The theory of the regulation of emotions (Gross, 2014) considers the processing of emotions in stressful situations from the viewpoints of reaction-focused expressive suppression (ES) and antecedent-focused cognitive reappraisal (CR; Gross, 2014; Gross & Levenson, 1993, 1997; John & Gross, 2004). While CR modifies an emotion that is still incompletely developed, ES acts in response to the emotion that is already fully experienced and modifies its behavioral expression (e.g., avoidance of verbal expression Gross, 2014). ES requires selective attentional processes that emphasize relevant information and suppress irrelevant information (Ochsner & Gross, 2005). Moreover, it affects the entire process of language production, from word finding to verbalization to suppress the expression of one’s emotional response (Gross, 2014; Gross & Levenson, 1997; Lindquist, 2017; Roche & Arnold, 2018). Individuals who actively suppressed their emotions through ES showed difficulties in their verbal fluency (Roche & Arnold, 2018), illustrating the negative impact of ES on communication.

## Development of a Process Model for Speechlessness

**Fig. 2:**
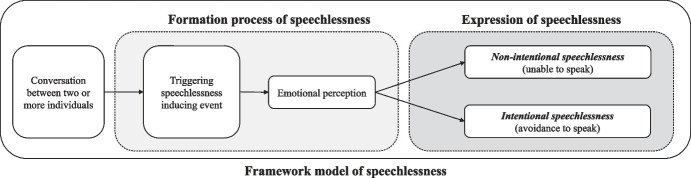
Framework model of speechlessness

Emotions and their processing and regulation in personally relevant contexts are key to the development of speechlessness, based on the reviewed literature and the described possible causes of speechlessness. The necessity of emotions in terms of their processing and/or regulation is made tangible by the term “meaningful emotions” (used in the following examples). The perception of meaningful emotions “(…) begins with an individual’s assessment of the personal meaning of some antecedent event” (Fredrickson, 2001, p. 218), which is in the context of relevance, value, or the individual’s own goal attainment process (see section above on *psychological flexibility*, *self-efficacy* and *Expectancy Violations Theory*) and can be described in various ways (Ekström et al., 2020; Park & Folkman, 1997). Meaningful emotions cannot be quantified because they are always subject to evaluation by the individual based on his or her own values, goals, preferences, etc. (Fredrickson, 2001). Considering these aspects, we constructed an overarching, processual framework of speechlessness, adapting the components of non-strategic (synonym: *non-volitional* or *non-intentional*) and strategic (synonym: *volitional* or *intentional*) speechlessness stated by Berger (2004) (Fig. 2).

The observed state of not speaking (phenomenon of speechlessness) is embedded in a framework, which describes the emergence process in two successive phases (see Fig. 2). The starting point of the process model is the conversational situation between two or more individuals. Within this phase, an event occurs that can trigger the formation of speechlessness. This event is associated with emotions (negative as well as positive) that are perceived consciously by the individual, and its awareness to the situation (cf. Table 3; see Fredrickson, 2001). Emotional perception and processing forms a core element in the emergence of speechlessness. Depending on the perceived emotions and their individual meaning (emotional valence depending on the situation), not speaking is manifested in the second phase of the process and is defined as *non-intentional* or *intentional speechlessness*. If the individual is overwhelmed by the perceived emotions and/or if these consist for example of fear, sadness or pain, the phenomenological consideration of the expressed speechlessness is based on a non-intentional, non-strategic speechlessness. If, on the other hand, the event triggering the speechlessness provokes a feeling of discomfort and a valence (meaning) of subsequent reactions which the individual connotes negatively and seeks to avoid, the phenomenological speechlessness to be observed is based on an intentional, strategic decision (intentional speechlessness).

The ‘emotional perception’ element (Fig. 2) can be seen as a mediator. Emotional processing is also a stage in the development of speechlessness that takes place within the individual and cannot be observed. Accordingly, emotional perception is a mediating variable that influences the outcome of speechlessness, which is specifically differentiated in this model. The present framework model does not correspond to a classical single mediator model (cf. MacKinnon, 2011), but integrates the mediating variable directly into the model.

***Example 1: Non-intentional speechlessness***: A young woman has her annual breast cancer screening. For her, this is a normal situation. No family history of the disease exists, nor are there any other predispositions that would increase the likelihood of a positive diagnosis. The examination is followed by a medical consultation. The doctor informs the young woman that the examinations indicate that there is a high probability that she has breast cancer. The doctor explains the next stages of treatment and inquires whether the patient has been adequately informed. However, the patient does not respond to the doctor’s question or the content he has explained. In this situation, the doctor sees a speechless patient. The young woman, on the other hand, is unable to answer or participate verbally in the conversation situation due to the current situation and the experienced meaningful emotions (e.g. fear, worry, panic) as well as a negatively violated expectation (violated expectation that no abnormalities were recorded in the screening examination). In this example, the medical conversation forms the initial situation (*conversation between two or more individuals*). The medical diagnosis embodies the *triggering speechlessness-inducing event*. The young patient’s *perception and processing of meaningful emotions* subsequently lead to the emergence of *non- intentional speechlessness*.

***Example 2: Intentional speechlessness***: A man is involved in a crime and is being investigated as a suspect. In the interrogation (*conversational situation*), the man is confronted with his deeds and encouraged to incriminate his accomplice in order to receive a reduced sentence (*triggering speechlessness inducing event*). On an emotional level, the criminal processes and evaluates the consequences of betraying his accomplice. Possibly the criminal regrets his deed and feels ashamed of it. Shame or guilt could be meaningful emotions for the criminal, which he tries to avoid expressing (e.g. *expressive suppression*). On the other hand, the man may be afraid to betray his accomplice because he might take revenge on him or his family (e.g. appearance of the emotion ‘fear’, which could be significant for the man). Instead of confessing, the criminal decides to remain silent due to the factors listed. To the people involved in the interrogation, the criminal appears speechless. However, this speechlessness is based on the decision of a conscious concealment in the form of silence. The emergence of speechlessness in this case follows an identical path similar to the first example, starting with a *conversational situation* (interrogation), a *triggering speechlessness inducing event* (severe judgment and possibility of confession), the *perception and processing of meaningful emotions* and potential consequences of the individual’s decision (a confession might lead to revenge by the accomplice, and this thought might frighten the man), and finally the resulting conscious decision to remain silent (*intentional speechlessness*).

Phenomenologically, *non-intentional* and *intentional* speechlessness are indistinguishable for the interlocutor. In order to differentiate between both forms, it is necessary to ask specific questions (e.g., in the form of survey instruments and/or qualitative interviews). At present, such survey instruments are not available. Table 4 provides an overview of potential factors that may have an influence on the emergence of non-intentional and intentional speechlessness.

**Table 4:** Categorization and definition

Form	Categorization	Potentially related emotions	References
Non-intentional speechlessness	non-voluntary, temporary difficulties in one’s own verbal expressions due to emotional overpowering	extreme emotions (e.g. fear, sadness, worry, joy)	(Bennett et al., 2020; Berger, 2004; Burgoon, 2015; Floyd et al., 1999; Koskinen et al., 2016; Lillrank, 2002; Lumley et al., 2011; Müller-Cyran, 2007)
Intentional speechlessness	conscious process of silence due to perceived and processed emotions with an accompanying subjective goal achievement	emotional discomfort (e.g. shame, embarrassment)	(Berger, 2004; Dimitrov, 2017; Kahn, 2019; Kummer et al., 2008)

In applying the model, it is important to note that the underlying concept of speechlessness must be consistent with a specific characteristic of non-speaking/silence. For example, if a person stutters, this is less likely to be considered speechlessness. Although the fluent flow of speech is disturbed, the clear intention of a verbal utterance is evident. However, if the individual shows an intention to say something (e.g. an open mouth), but no or only fragmentary sounds come out, this should be taken as an indicator of possible non-intentional speechlessness. In addition, the average duration of speech acts in conversational exchanges can be used as an orientation. The production of a single word takes 600 milliseconds, that of a sentence 1,500 milliseconds and the average response time 2 s (Levinson, 2016). The estimated duration of speechlessness of the interviewed participants in Berger’s (2004) study is indicated as a mean of 26.36 (SD = ± 299.64) seconds (range: 0.03–4.320 min). Exceeding the duration of > 2 s can be seen as an indicator of the occurrence of speechlessness.

## Discussion

In this work, speechlessness is not considered as a new form of psychopathology. Speechlessness is rather an unusual (more specific: unique, exceptional or peculiar) reaction to adversary or extraordinary circumstances. From a scientific point of view, speechlessness is a phenomenon that has hardly been considered so far, but is a common theme in public. The aim of the present study was to examine speechlessness as an “observable phenomenon of non-speaking” from a psychological perspective. Within this objective, the identification of possible factors contributing to the emergence of the phenomenon of speechlessness was also conducted.

The results presented in this study are based on the comprehensive preliminary work of Berger (2004) on the topic of speechlessness. The need for a psychological definition of the phenomenon of speechlessness becomes clear, considering the relevance of the topic in public and mental health care for individuals with anxiety, depression, or post-traumatic health problems, or health care for patients with chronic or life-threatening illnesses. Considering the original scope of literature presented above (cf. Figure 1), a large number of studies on the topic of speechlessness were omitted due to a psychopathological or physiological background, despite clearly defined search terms. The need to explore speechlessness from a phenomenological perspective is supported by the limited number of sources finally sighted.

After considering the reviewed literature (Berger, 2004; Fabiane & Corrêa, 2007; Kahn, 2019; Koskinen et al., 2016; Kummer et al., 2008; Lillrank, 2002; Müller-Cyran, 2007), it can be seen that intense emotions of an individual, which includes the individual’s perception and processing (e.g. perceived fright of of the environment of an intensive care unit; cf. Fabiane & Corrêa, 2007), are directly related to speechlessness. However, considering the search terms used and the included studies, none of these were identified by a search term related to “emotions” (cf. Table 2). The relevance of emotions is directly derived from the results recorded by the authors.

Emotions not only constitute an individual’s subjective experience, but rather influence our behavior to act (or not to act) (Gross, 2015; Mauss et al., 2005). This relationship is also evident in the phenomenon of speechlessness. Emotions experienced by the individual promote socially appropriate behavior (Averill, 1980) and inform us about the intentions of those around us (Fridlund, 1994). The goal of emotion regulation, an active adjustment of the course of perceived emotions (Gross et al., 2011), can occur consciously and unconsciously in this process (Gross, 2015; Gyurak et al., 2011). The higher-order process of emotion perception, processing, and regulation turns out to be one of the most important elements of human behavior and social interaction (Andersen & Guerrero, 1996; Lopes et al., 2005). Accordingly, emotions have been assigned a gatekeeping position in the processual model of the phenomenon of speechlessness. The process model developed from the literature, concepts and theories discussed represents a simplified explanatory approach to the development and observed form of speechlessness. The model uses a path which consists of the individual and necessary components for the emergence of speechlessness. A comparable model is the process of emotion regulation according to Gross (1998, 2014, 2015) or the attention-appraisal model (Preece et al., 2017). The observable phenomenon of speechlessness must be preceded by a conversational situation between at least two or more people and an event (e.g., topic of conversation, social interaction, or similar). The latter triggers a process of emotional perception and processing in the individual. As a result of this process, the individual is ‘unable to speak’ due to a loss of words (cf. Berger, 2004) or ‘will not speak’ due to internal/external factors (cf. Kurzon, 2020). This nuance forms the primary result of the present work and justifies a continuing, intensive examination of the phenomenon of speechlessness. The model we have developed also allows us to classify potential situations of speechlessness. In particular, considering the average duration of responses within a conversational situation (~ 2 s; cf. Levinson, 2016), the model provides a complementary way to interpret the reasons for exceeding this duration. Furthermore, the model can be seen as a first step or guide for further exploration of the research field of speechlessness.

## Limitations, Conclusion and Future Work

This paper does not claim to be able to describe the phenomenon of speechlessness in its entirety. Nor do we as authors claim that our model and the division of non-speaking into an intentional and a non-intentional dimension reflect ‘reality’. Rather, the motivation is to look at the phenomenon of speechlessness through the lens of psychological inquiry. Despite the diverse and theoretically grounded search terms, it cannot be ruled out that relevant research was excluded from the systematic review. Another possible limitation is the deduction of Berger’s (2004) understanding of speechlessness to the literature we analyzed and discussed above. Based on the reasons for speechlessness mentioned in the foundational work, a corresponding interpretation of the forms of speechlessness described in the researched sources was made.

From an observational perspective, non-intentional and intentional speechlessness are indistinguishable, as the speechless individual does not express himself verbally in either form. People who are frequently confronted with such situations (e.g. doctors, therapists, legal officials) may be able to better distinguish between deliberate/strategic silence (intentional speechlessness) and non-strategic silence (non-intentional speechlessness). It should also be noted that intentional silence, which has been added to the work on intentional speechlessness in this paper, is a separate aspect of research in the literature (cf. search term “*strategic silence*”; cf. Dimitrov, 2017). It should be noted that the literature identified in the present work focuses primarily on the influence of negative emotions on the development of speechlessness. However, it can be assumed that positive emotions can also contribute to the development of speechlessness (cf. positive expectation violation, EVT; Burgoon, 2015), for example, when a person unexpectedly wins the lottery (cf. Berger, 2015).

The (new) approach of our work is to describe speechlessness in a processual framework with explicit inclusion of emotional perception and processing. The observable phenomenon of speechlessness is thus formulated under the aspects of an intrinsically motivated silence or an externally conditioned “inability to speak”. This may seem incoherent at first glance, but our analysis shows that both forms of the observer’s speechlessness are inseparable and have been studied together. This is particularly evident in the simultaneous use of the terms “silence” and “speechlessness” (cf. Kurzon, 2007). The model’s claim that silence is based on an intentional process also needs to be tested empirically. Furthermore, silence should be examined in more detail in further studies using the developed framework model.

The systematic literature search based on theory-guided search terms did yield a small number of sources with a description and/or definition of speechlessness. However, these sources all contained clear parallels to Berger’s (2004) preliminary work in their description. The exploratively derived framework, based on the forms of speechlessness described in the discussed literature, is characterized by its low complexity and multiple applicability in conversational situations. To the authors’ knowledge, it is also the first framework model for a phenomenological description of speechlessness. At the same time, the present work underlines the relevance of researching the field of speechlessness. Future studies can adopt the framework model and use it to investigate the relationships between (unintentional and intentional) speechlessness and emotional perception and processing. In addition, researchers could apply the model to different conversational situations in order to derive relevant questions and hypotheses. Furthermore, the research field around the phenomenon of speechlessness should be explored through further work. Besides implementing the framework model, applying specific survey instruments would be useful for this purpose.

From our perspective, further exploration of the phenomenon of speechlessness is valuable only against the normative background of meaningful emotions and situations. In addition, future explorative/empirical studies could also refer to existing (alexithymia, aphasia, etc.) or new (e.g., alexinomia) concepts/constructs related to speechlessness or silence in order to expand the understanding and support of the presented concept. The approach to speechlessness embodied in this work and the processual framework model derived from it has the long-term goal of distinguishing between the trivial and metaphorical use of speechlessness as an expression of empathy or concern (e.g., expressing subjective regret and concern over the violation of human rights of refugees in conflict regions) and actual meaningful speechlessness for individuals. We look forward to future work on the phenomenon of speechlessness.

## Data Availability

All data (literature sources) included in this publication are publicly available and are cited accordingly. No additional data were collected as part of this publication, and therefore no data are available.

## Footnotes

1 Anatomically, Wernicke’s area corresponds to the superior temporal gyrus, the supramarginal gyrus, and the angular gyrus.

2 Anatomically, Broca’s area corresponds to the inferior frontal gyrus.

3 An interlocutor is a person who takes part in a dialog or conversation.

4 Omertà implies the ‘prohibition’ of cooperation by Mafia members with investigating authorities, the government and outsiders. The term originates from the Italian Mafia and is still used today in southern Italy. Individuals who break the code must expect sanctions against themselves or their families, primarily in the form of physical attacks (Oxford English Dictionary, 2022; Pitrè, 1889).

5 As the results of this study (Berger, 2004) have already been discussed in detail in the previous section, the listing/interpretation will not be repeated.

6 The term “meaningful emotions” embodies the subjective perception of the relevance and usefulness of these emotions in the context of one’s own goal attainment, values, attitudes, or personal identity. A detailed definition of the term can be found in the section “Development of a Process Model for Speechlessness”.
